# Zoledronic Acid-Related Hypocalcemia and Renal Dysfunction in Oncology Patients: A Tertiary Care Experience

**DOI:** 10.7759/cureus.98055

**Published:** 2025-11-28

**Authors:** Muhammad Ahsan Jamil, Muhammad Awais Majeed, Muzammil Ali, Hiba Asif, Maryam Imran, Muhammad Imran, Saad Aslam, Ovais Qureshi, Noor Ul Ain, Shehar Bano, Naamaan Sadique, Farzan Ali, Sameen Bin Naeem

**Affiliations:** 1 Medical Oncology, Shaukat Khanum Memorial Cancer Hospital and Research Centre, Lahore, PAK; 2 Oncology, Shaukat Khanum Memorial Cancer Hospital and Research Centre, Lahore, PAK; 3 Internal Medicine, Shaukat Khanum Memorial Cancer Hospital and Research Centre, Lahore, PAK; 4 Hematology, Shaukat Khanum Memorial Cancer Hospital and Research Centre, Lahore, PAK

**Keywords:** cancer patients, creatinine, hypocalcemia, nephrotoxicity, zoledronic acid

## Abstract

Background

Zoledronic acid, a bisphosphonate commonly used to manage bone metastases and malignancy-associated hypercalcemia in cancer patients, has proven clinical efficacy in preventing skeletal-related events. However, its use is associated with adverse effects, particularly hypocalcemia and renal dysfunction. Despite its widespread application, data from local cancer populations remain limited, which may impact the development of region-specific risk mitigation protocols. This study was conducted to assess the frequency of hypocalcemia and elevated serum creatinine levels in cancer patients treated with zoledronic acid at a tertiary care cancer hospital.

Methodology

This retrospective observational study was conducted at the Department of Oncology, Shaukat Khanum Memorial Cancer Hospital and Research Centre, Lahore, Pakistan. Medical records of 400 adult cancer patients treated with intravenous zoledronic acid between January 1 and December 31, 2024 were reviewed. After applying exclusion criteria, including baseline hypercalcemia and chronic kidney disease, 243-300 patients were included in the final analysis for calcium and creatinine outcomes. Data on demographics, baseline, and post-treatment serum calcium and creatinine levels were collected and analyzed using SPSS. Paired samples t-tests were used to assess statistical differences, with p<0.05 considered significant.

Results

Following treatment, the mean serum creatinine level rose from 0.714 mg/dL to 1.572 mg/dL (mean increase: 0.86 mg/dL, p<0.001), indicating a statistically and clinically significant decline in renal function. Similarly, mean corrected serum calcium dropped from 9.41 mg/dL to 8.52 mg/dL (mean decrease: 0.89 mg/dL, p<0.001), confirming a significant hypocalcemic effect. Approximately 29.4% of patients developed post-treatment creatinine levels >1.3 mg/dL, and 2.0% experienced calcium levels <7 mg/dL.

Conclusion

Zoledronic acid was associated with significant post-treatment increases in serum creatinine and decreases in calcium levels in cancer patients. These findings reinforce the need for routine renal and calcium monitoring, along with appropriate supplementation, to improve treatment safety and patient outcomes.

## Introduction

Zoledronic acid, a nitrogen-containing bisphosphonate, is extensively used in oncology for the management of cancer-associated bone disease. It effectively prevents skeletal-related events such as pathological fractures, spinal cord compression, and the need for radiation or surgery to bone in patients with bone metastases [1]. This therapeutic benefit is particularly significant in advanced cancers such as breast, prostate, lung, and multiple myeloma, where osseous metastasis is prevalent [2,3]. Despite its clinical utility, zoledronic acid is associated with notable adverse effects, primarily hypocalcemia and renal dysfunction, which necessitate vigilant monitoring [4,5].

Hypocalcemia following zoledronic acid therapy is linked to the drug's mechanism of action: inhibition of osteoclast-mediated bone resorption, which transiently lowers serum calcium levels [6]. The incidence of hypocalcemia in patients receiving bisphosphonates has been reported in the range of 10% to 25%, influenced by factors such as calcium and vitamin D status, cancer type, and pre-existing conditions [7,8]. Renal toxicity, another well-documented side effect, may present as elevated serum creatinine or, in severe cases, acute kidney injury [9]. The risk of nephrotoxicity is particularly elevated in patients with dehydration, pre-existing renal impairment, or concomitant use of nephrotoxic medications [10,11].

Although several international studies have investigated the frequency and predictors of these adverse events [12-14], data from South Asian or lower-middle-income country settings remain sparse. Regional differences in patient demographics, comorbidities, dietary calcium intake, and access to laboratory monitoring could alter the risk profile of these complications. Furthermore, many international studies are conducted in controlled environments or among highly selected patient populations, limiting their generalizability to real-world settings [15]. Without region-specific data, it is challenging to formulate effective monitoring protocols or establish evidence-based thresholds for risk mitigation.

The primary objective of this study was to assess the frequency of hypocalcemia and deranged serum creatinine levels in adult cancer patients receiving zoledronic acid therapy.

## Materials and methods

Population

The study population consisted of adult patients (≥18 years) with confirmed malignancies treated at the Department of Oncology, Shaukat Khanum Memorial Cancer Hospital and Research Centre (SKMCH&RC), Lahore, Pakistan. All patients who received at least one dose of zoledronic acid for malignancy-associated bone disease between January 1, 2024, and December 31, 2024, were eligible.

Exclusion criteria

Exclusion criteria were baseline hypercalcemia (serum calcium >10.5 mg/dL); pre-existing chronic kidney disease (serum creatinine >1.4 mg/dL in men, >1.2 mg/dL in women); incomplete clinical records; patients who did not complete the prescribed course of therapy; and patients on nutritional supplements.

Intervention

The intervention of interest was zoledronic acid administration for the treatment of bone metastases or malignancy-associated bone disease. Baseline and follow-up laboratory values of serum calcium and serum creatinine were collected around the zoledronic acid infusion.

Comparison

Patients served as their own control, with outcomes compared between baseline measurements (most recent serum calcium and creatinine levels prior to infusion) and post-treatment measurements (lowest calcium and highest creatinine values recorded within 30 days after infusion); no external comparison group was used due to the retrospective observational design.

Outcomes

The primary outcomes were frequency of hypocalcemia following zoledronic acid therapy and incidence of renal function derangement, assessed by changes in serum creatinine within 30 days of treatment.

Study design

This was a retrospective observational study conducted over one year. Ethical approval was obtained from the SKMCH&RC Institutional Review Board (IRB). Given the retrospective design and use of anonymized data, the requirement for informed consent was waived.

Patient data were extracted from electronic medical records and the oncology pharmacy database. Variables collected included demographics, cancer type, comorbidities, baseline labs, and post-treatment lab changes. Data confidentiality was maintained through anonymization and a secure, password-protected research database with routine validation checks.

Statistical methods

All statistical analyses were conducted using IBM SPSS Statistics for Windows, Version 26 (Released 2018; IBM Corp., Armonk, New York, United States). Descriptive statistics were used to summarize demographic and clinical variables. Paired samples t-tests were applied to evaluate changes in serum calcium and creatinine levels pre- and post-treatment. A p-value of <0.05 was considered statistically significant.

## Results

Patient cohort and baseline characteristics

The study included 300 adult cancer patients who received zoledronic acid. Due to incomplete records, the final analytical cohorts for creatinine and calcium assessments consisted of 243 and 239 patients, respectively. The demographic and clinical profiles of the patient population are detailed in Table 1.

**Table 1:** Baseline Demographics and Clinical Characteristics of the Patient Cohort (N=300). Data are presented as n (%). Percentages may not sum to 100 due to rounding. a. Other cancers include head and neck cancer (n=2), renal cell cancer (n=2), and colorectal cancer(n=1). N, total number of patients; n, number of patients in a subgroup.

Characteristic	Patients (N=300)
Gender	
Female	235 (78.3%)
Male	65 (21.7%)
Primary Cancer Diagnosis	
Breast cancer	245 (81.7%)
Prostate cancer	44 (14.7%)
Multiple myeloma	6 (2.0%)
Other cancers	5 (1.7%)

Biochemical outcomes following zoledronic acid administration

**Figure 1:**
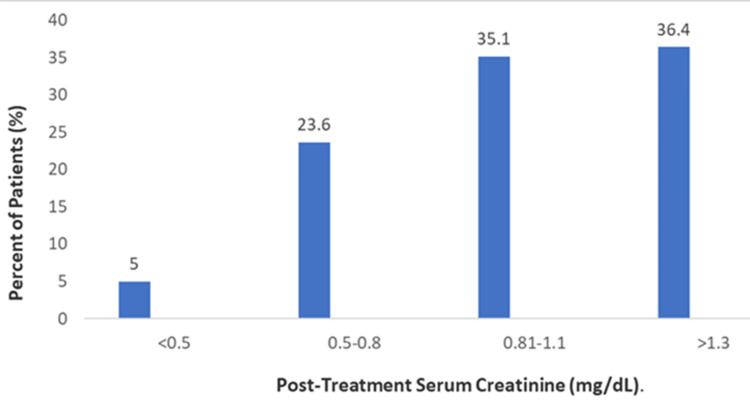
Distribution of Post-treatment Serum Creatinine Levels. This chart illustrates the percentage of patients within defined serum creatinine categories following the administration of zoledronic acid (n=242).

A notable deterioration in renal function was observed post-treatment. The proportion of patients with serum creatinine levels exceeding 1.3 mg/dL increased substantially, accounting for 29.4% of the cohort after infusion. This shift toward higher creatinine values is illustrated in Figure 1, and a detailed categorical breakdown of pre- and post-treatment levels is provided in Figure 1 and Table 2.

**Table 2:** Frequency Distribution of Serum Creatinine and Calcium Categories Before and After Zoledronic Acid Administration. Data are presented as n (%). Percentages are calculated based on the total number of patients (N) with available data for each specific analysis, as indicated in the column headers.

Laboratory Parameter	Baseline n (%)	Post-treatment n (%)
Serum Creatinine (Normal Value 0.4 - 1.1 Mg/dl)	(N=243)	(N=242)
<0.5	56 (23.0%)	12 (5.0%)
0.5-0.8	115 (47.3%)	57 (23.6%)
0.81-1.3	62 (25.5%)	85 (35.1%)
>1.3	10 (4.1%)	88 (36.4%)
Serum Calcium (8.5-10.5 mg/dL)	(N=241)	(N=229)
<7.0	0 (0.0%)	6 (2.6%)
7.0-9.0	62 (25.7%)	165 (72.1%)
9.1-11.0	167 (69.3%)	57 (24.9%)
>11.0	12 (5.0%)	1 (0.4%)

**Figure 2:**
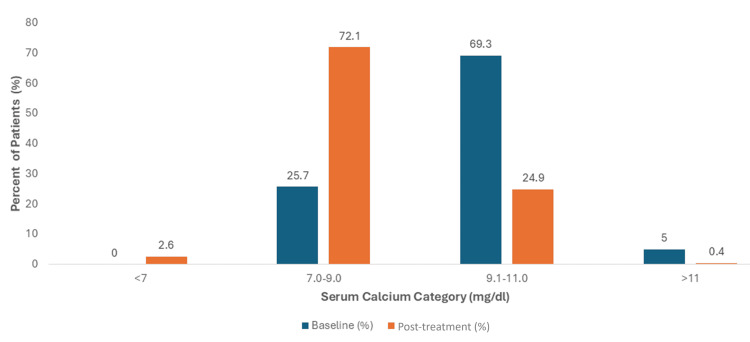
Distribution of Serum Calcium Categories Before and After Zoledronic Acid Treatment (mg/dL). This clustered bar chart compares the percentage of patients in each serum calcium category at baseline (n=241) versus post-treatment (n=229)

In parallel, a significant hypocalcemia trend was evident following treatment. The data revealed a pronounced shift toward lower serum calcium concentrations, with a clinically significant minority (2.0%) of patients developing severe hypocalcemia (levels <7.0 mg/dL). The distribution of patients across different calcium categories before and after treatment is compared in Figure 2.

Statistical analysis of treatment effects

Paired samples t-tests confirmed the clinical observations. In the 243 patients with complete creatinine data, the mean serum level increased from 0.714 mg/dL at baseline to 1.572 mg/dL post-infusion. This mean increase of 0.86 mg/dL (95% CI: 0.67 to 1.05) was statistically significant (t(242) = 8.778, p < 0.001).

**Figure 3:**
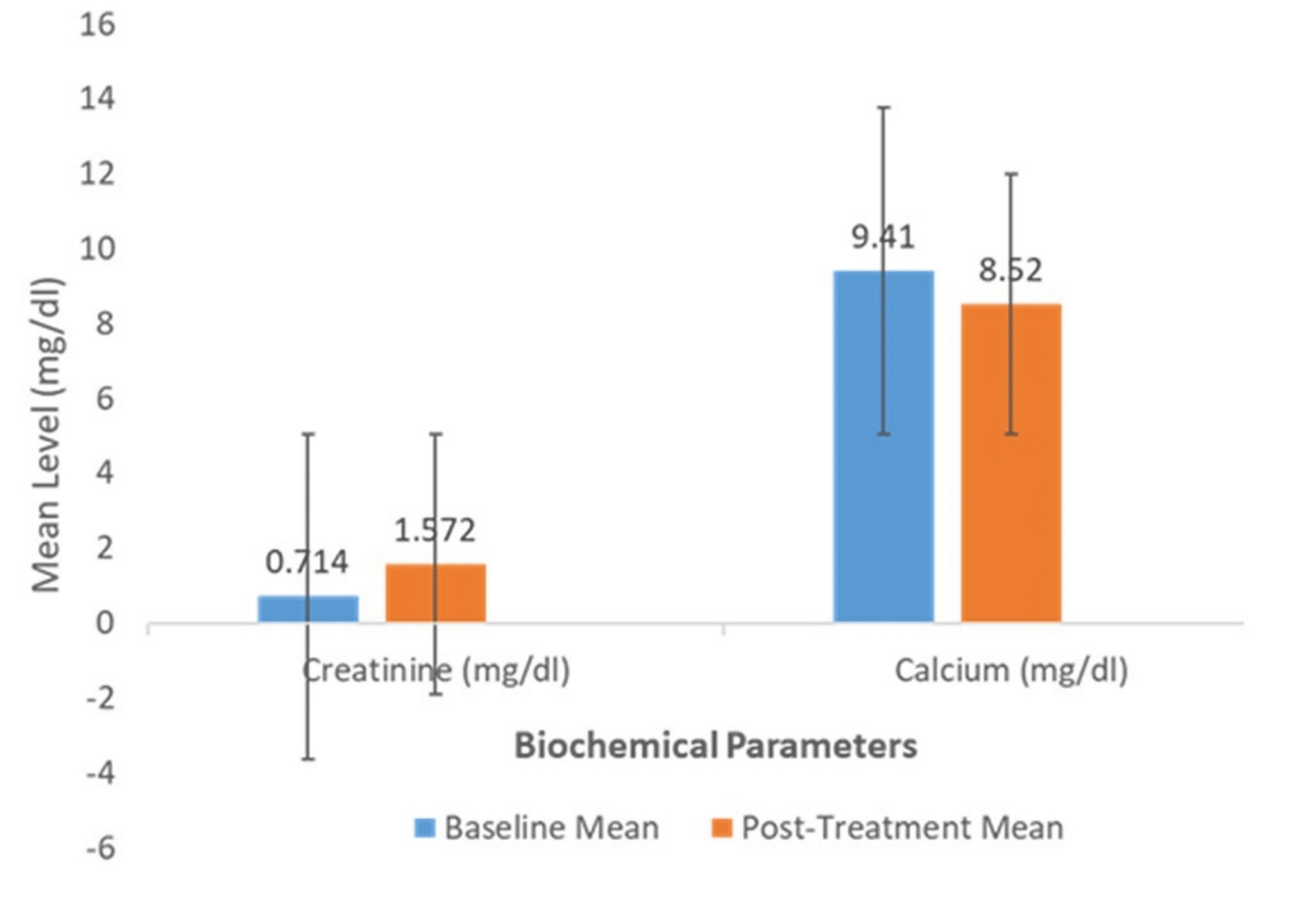
Comparison of Mean Serum Creatinine and Calcium Levels Before and After Zoledronic Acid Treatment. This bar chart displays the mean serum creatinine (n=243) and mean corrected serum calcium (n=239) at baseline versus post-treatment, with the X-axis representing the treatment timeline (pre-treatment and post-treatment). Error bars represent 95% confidence intervals. p < 0.001 for both comparisons

Consistent with this, the analysis of 239 patients with complete calcium data showed a mean decrease from 9.41 mg/dL to 8.52 mg/dL. The mean reduction of 0.89 mg/dL (95% CI: 0.76 to 1.03) was also highly significant (t(238) = 12.991, p < 0.001). These comparative findings are summarized in Figure 3, with detailed statistical outcomes presented in Figure 3 and Table 3.

**Table 3:** Paired Samples t-Test Results for Changes in Biochemical Markers. A positive mean difference indicates an increase from baseline; a negative mean difference indicates a decrease from baseline. Statistical significance was determined using a paired samples t-test. N, number of paired samples; CI, confidence interval; df, degrees of freedom.

Biochemical Marker	N	Mean Difference (95% CI)	t-value (df)	p-value
Serum Creatinine (mg/dL)	243	0.86 (0.67 to 1.05)	8.778 (242)	<0.001
Serum Calcium (mg/dL)	239	-0.89 (-1.03 to -0.76)	-12.991 (238)	<0.001

## Discussion

This study aimed to assess the frequency of renal dysfunction and hypocalcemia in cancer patients treated with zoledronic acid at a tertiary care center. The findings confirm that this therapy is associated with a statistically significant decline in renal function and a potent hypocalcemia effect, even in a cohort screened for pre-existing kidney disease. The results underscore the importance of vigilant biochemical monitoring as a standard of care.

The observed rise in serum creatinine is consistent with the well-documented nephrotoxic potential of bisphosphonates [16,17]. Notably, a substantial proportion of our patients experienced a decline in renal function despite having normal baseline creatinine levels, suggesting that the risk is not limited to those with pre-existing renal impairment. This finding reinforces the need for universal caution and may indicate that standard creatinine thresholds do not fully capture the at-risk population.

In contrast, the 2.0% incidence of severe hypocalcemia in our study is lower than the rates reported in some earlier literature, which ranged from 9% to 35% [16,18,19]. This discrepancy could be attributed to an evolution in clinical practice, particularly the now-widespread implementation of proactive calcium and vitamin D supplementation protocols. However, the risk is clearly not eliminated, as our findings align with more recent work showing that hypocalcemia remains a pertinent clinical issue, even with supplementation [20]. The significant mean drop in serum calcium observed in our cohort is a direct reflection of the drug's mechanism of action-powerful inhibition of osteoclast-mediated bone resorption.

The molecular knowledge of zoledronic acid-induced renal injury has advanced significantly over the last decade, with increasing evidence pointing to direct tubular toxicity caused by intracellular drug accumulation in renal proximal tubular cells [21,22]. This cellular absorption disrupts the mevalonate pathway and subsequently impairs protein prenylation, culminating in tubular cell death and functional degradation [23,24]. This process frequently shows up in patients as an increase in creatinine levels without any symptoms. However, there have been severe cases when patients receiving fast infusions or not drinking enough water have developed acute tubular necrosis [25,26]. Individual patient characteristics, such as baseline renal reserve, concomitant nephrotoxic medicines, and hydration state, seem to influence the extent of renal injury seen in clinical practice [27]. These results highlight the necessity of individual risk evaluation and the adoption of renal protective measures, including maintaining sufficient hydration, prolonging infusion durations to a minimum of 15 minutes, and refraining from the concurrent administration of additional nephrotoxic substances [28].

Similarly, the drug's powerful inhibition of farnesyl pyrophosphate synthase within osteoclasts results in a hypocalcemic impact because it disrupts the mevalonate pathway, which is required for osteoclast survival and function [29,30]. This technique, although therapeutically advantageous in diminishing skeletal morbidity, induces a temporary yet clinically substantial disturbance in calcium homeostasis that may endure for several weeks following infusion. The extent of calcium reduction seems to be affected by initial calcium levels, the sufficiency of vitamin D reserves, and the level of osteoclast activity associated with tumor load in bone [2]. Comprehending these molecular pathways substantiates the need for slow infusion techniques and assertive calcium-vitamin D supplementation tactics that are currently regarded as standard practice in the majority of oncology centers. Even with these preventative steps, breakthrough hypocalcemia can still happen, especially in patients with a lot of bone involvement or those who don't follow their supplementing plan. This shows that we need to keep an eye on things even when prophylactic measures are in place.

The primary strengths of this study include its relatively large sample size and the use of a paired-samples statistical design, which enhances the internal validity of the findings. However, the study has some limitations inherent to its retrospective design. Furthermore, we could not assess other important confounders, including hydration status, concurrent nephrotoxic medications, and baseline vitamin D levels.

The clinical implications of this study are direct and actionable. It serves as a stark reminder that zoledronic acid administration requires a commitment to rigorous pre- and post-infusion monitoring of renal and calcium markers. Future research should focus on prospective, multicenter studies to develop and validate risk stratification models. Such models could help identify patients at the highest risk for adverse events, potentially allowing for dose adjustments or the selection of alternative therapies to balance therapeutic efficacy with patient safety.

## Conclusions

This study demonstrated that zoledronic acid therapy in cancer patients is associated with a significant increase in serum creatinine levels and a notable reduction in serum calcium concentrations. These findings underscore the drug's potential to induce renal dysfunction and hypocalcemia, even in patients without pre-existing renal impairment. The results highlight the importance of routine biochemical monitoring before and after treatment to ensure early detection and management of these adverse effects.

For clinical decision-making, the study reinforces the need for individualized assessment of renal function and calcium status prior to initiating zoledronic acid, along with the implementation of preventive strategies such as hydration and supplementation protocols. Future prospective studies are recommended to validate these findings across broader populations and to explore predictive risk models that can aid in identifying vulnerable patients. Incorporating such risk assessment into treatment planning may enhance the safety and efficacy of bisphosphonate use in oncology practice.
